# Effect of *GRM7* polymorphisms on the development of noise-induced hearing loss in Chinese Han workers: a nested case-control study

**DOI:** 10.1186/s12881-017-0515-3

**Published:** 2018-01-05

**Authors:** Peipei Yu, Jie Jiao, Guoshun Chen, Wenhui Zhou, Huanling Zhang, Hui Wu, Yanhong Li, Guizhen Gu, Yuxin Zheng, Yue Yu, Shanfa Yu

**Affiliations:** 1 Department of Occupational and Environmental Health Hygiene, College of Public Health, Zhengzhou University, Zhengzhou, Henan People’s Republic of China; 2 Henan Provincial Institute for Occupational Health, No.3, Kangfu Mid. St, Zhengzhou, 450052 People’s Republic of China; 3 Wugang Institute for Occupational Health, Wugang, Henan People’s Republic of China; 4 College of Public Health, Qingdao University, Qingdao, Shandong People’s Republic of China; 5 National Institute of Occupational Health and Poison Control, Chinese Center for Disease Control and Prevention, Beijing, People’s Republic of China

**Keywords:** *GRM7*, Noise-induced hearing loss, Polymorphism

## Abstract

**Background:**

Noise-induced hearing loss (NIHL) is a complex, irreversible disease caused by the interaction of genetic and environmental factors. In recent years, a great many studies have been done to explore the NIHL susceptibility genes among humans. So far, high powerful detections have been founded that genes of potassium ion channel genes (*KCNQ4* and *KCNE1*), catalase (*CAT*), protocadherin 15 (*PCDH15*), myosin 14 (*MYH14*) and heart shock protein (*HSP70*) which have been identified in more than one population may be associated with the susceptibility to NIHL. As for metabolic glutamate receptor7 gene (*GRM7*), a lot of researches mainly focus on age-related hearing loss (ARHL) and the results have shown that the polymorphisms of *GRM7* are linked to the development of ARHL. However, little is known about the association of *GRM7* and the susceptibility to NIHL. Therefore, the aim of this study was to explore the effect of *GRM7* polymorphisms on the susceptibility to NIHL.

**Methods:**

A nested case-control study based on the cohort in a Chinese steel factory was implemented in 292 cases and 584 controls matched with the same sex, the age difference ≤ 5 years old, the same type of work, duration of occupational noise exposure ≤2 years. Five single nucleotide polymorphisms (SNPs) of *GRM7* were gained through selecting and genotyping SNPs. Conditional logistic regression analysis was used to assess the main effect of *GRM7* polymorphisms on the susceptibility to NIHL and the gene-by-environment interaction. Furthermore, the gene-by-gene interactions were analyzed by generalized multiple dimensionality reduction (GMDR).

**Results:**

This research discovered for the first time that the mutant allele C in rs1485175 of the *GMR7* may decrease individuals’ susceptibility to NIHL. The interaction between rs1485175 and cumulative noise exposure (CNE) at high level was found after the stratification according to CNE (*p*/*p*_*bon*_ = 0.014/0.007, OR = 0.550, 95% CI: 0.340–0.891). Permutation test of GMDR suggested that rs1920109, rs1485175 and rs9826579 in *GRM7* might interact with each other in the process of developing NIHL (*p* = 0.037).

**Conclusions:**

The results suggest that the mutant allele C of rs1485175 in *GRM7* may reduce the susceptibility to NIHL in Chinese Han population.

**Electronic supplementary material:**

The online version of this article (10.1186/s12881-017-0515-3) contains supplementary material, which is available to authorized users.

## Background

With the widely spread of industrialization in the world, noise exposure is becoming more common in the industrial settings. This phenomenon is more noteworthy in developing countries. Nowadays, hearing loss due to occupational noise exposure is still an intractable problem for both developing and developed countries, and a lot of studies on noise are mainly focused on occupational NIHL [1]. World Health Organization studies show that occupational NIHL, as the second occupational health hazard except unintentional injuries, accounts for 16% of occupational chronic diseases and 19% of the loss of health life induced by noise exposure in work place [2, 3]. About 22 million U.S. workers are exposed to harmful noise level in the working environment every year and NIHL is one of the most common occupational diseases in the United States [4, 5]. In China, occupational NIHL is the third most harmful occupational disease, accounting for one-sixth in all of the annual increased occupational diseases recently [6].

It has been well known that NIHL is a complicated disease caused by the interaction of genetic and environmental factors. The environmental factors, such as noise exposure, organic solvents, ototoxic drugs, heat, vibrations, smoking and health relative factors (hypertension, high cholesterol, pigmentation) and so on, all have essential effect on the progress of NIHL and a lot of work on preventing the harmful effect induced by these factors has been done. However, studies on human genetic factors which may be associated with the susceptibility to NIHL are relatively rare [7]. Researches on the knockout mice, for example *Pjvk*^*−/−*^ [8], *PMCA2*^*+/−*^ [9], *P2RX2*^*−/−*^ [10], *CDH23*^*+/ -*^[11], *SOD*^*−/−*^ [12], *GPX1*^*−/−*^ [13], have indicated that the gene deficiency mice are more susceptible to NIHL. Current studies have shown that the polymorphisms of potassium ion channel genes (like *KCNQ4* and *KCNE1*) [14, 15], catalase (*CAT*), protocadherin 15 (*PCDH15*), myosin 14 (*MYH14*) [16] and heart shock protein (*HSP70*) [17] detected in different populations are significantly related to the development of NIHL. In addition, studies conducted by our research group has also found that heart shock protein (*HSP70*) [18], eye absent homolog 4 (*EYA4*) [19], suggestive POU-domain transcription POU4F3 and Grainyhead-like2 (*GRHL2*) [20] may be associated with the susceptibility to NIHL.

Up to now, when it comes to *GRM7*, previous studies have shown that *GRM7* polymorphisms are associated with the susceptibility to hearing loss in the elderly [21–23], but the relationship between GRM7 polymorphisms and NIHL susceptibility remains to be further validated. At present, many researches have shown that glutamate is the main excitatory neurotransmitter in the transmission of inner hair cells and type I spiral ganglion neurons [24, 25]. High concentration of glutamate is neurotoxic, which has been known to be associated with NIHL [21, 26]. Metabotropic glutamate receptor 7 (mGluR7) encoded by *GRM7* can reduce excessive glutamate release in the synaptic compartments to maintain their normal physiological concentration [21]. High-level noise exposure leads to the excessive release of glutamate from the hair cells to the synaptic cleft and the high concentration of glutamate overstimulates the postsynaptic cells or dendrites, causing them excitatory poisoning with the feature of swelling [27–29].

Hence, we hypothesize that *GRM7* polymorphisms may have an effect on the development of NIHL. Then, we screen single nucleotide polymorphisms (SNPs) of *GRM7* that may be related to the susceptibility of NIHL and carry out a nested case-control study in the occupational populations to analyze the relationship between *GRM7* polymorphisms and NIHL.

## Methods

### Subjects

A dynamic cohort was established in a big steel factory among lasting noise exposure workers On January 1, 2006. Occupational health examinations, hearing tests and questionnaire surveys were carried out among the 6886 works selected into the cohort in the first year. And then noise, heat, toxic and hazardous substances in the work environment were monitored and measured every year. Health examination and hearing test were conducted every two years for the studying population. Up to December 31, 2015, there were 6297 subjects completed more than twice health examinations and hearing tests and 817 subjects finished only once. In the process of follow-up, 559 subjects joined in the research and 331 subjects were loss to follow-up because of resignation or being transferred to other positions.

In the cohort, there were 9 individuals with a history of being an airman, 76 with a history of being an artillerist, 53 with a history of head trauma, 3 with a history of blast exposure hearing damage, 10 with a history of eardrum perforation, 1 with a history of taking ototoxic drugs, 32 with a familial history of deafness, 15 with a history of rubella, 4 with a history of Meniere’s syndrome, all of which were excluded in the case and control selecting.

This research based on the cohort study with 1 case to 2 controls matched. The case group (hearing loss group) and the control group were selected with the level of occupational noise exposure ≥80 dB(A) and the time of accumulated occupational noise exposure ≥3 years. The inclusion of cases was that binaural average hearing threshold levels (HTLs) in high frequencies (3 kHz,4 kHz,6 kHz) ≥ 40 dB(A). The control group matched with the same sex, the age difference ≤ 5 years old and the same type of work, duration of occupational noise exposure ≤2 years and was selected according to the HTL of any one ear in linguistic frequencies (0.5 kHz, 1 kHz, 2 kHz) < 25 dB(A) and average binaural HTL in high frequencies <35 dB(A). Finally, there were 292 cases and 584 controls entering into this study.

The study was approved by the Ethics Committee of Henan Provincial Institute for Occupational Health (Ethical approval no.: 2,013,003) and informed consent was signed by all study participants or their agents.

### Epidemiological survey

A combinative method of investigators interviewing and respondents actively reporting were used to collect information. Investigators had been professionally trained in advance. The questionnaire of this study mainly included the following aspects: (1) demographic characteristics: such as age, gender, date of birth, educational level, etc. (2) professional history: such as type of work, noise exposure duration in noise setting, environmental noise exposure level, etc. (3) living habits: such as, whether smoking and daily smoking levels, whether drinking and daily alcohol consumption, high-fat food intake, etc. (4) previous history of diseases affecting hearing: such as, ear trauma, tinnitus, sudden deafness, hypertension, etc. (5) history of ototoxic drug use, such as: aminoglycosides or vancomycin antibiotics, containing cisplatin and other anti-tumor drugs, containing arsenic and other heavy metal drug use history and so on. The detailed questionnaire was offered in the supplementary file [see Additional file 1].

Smokers were those smoking at least one cigarette every day and more than 6 months, otherwise, they were regared as non-smokers. The criterion for judging drinkers was that subjects drank at least once per week more than 1 year, if not, they were thought as non-drinkers.

### Hearing test and ear examination

Before the examination, all the subjects were required to leave the occupational noise environment for at least 12 h. 216 audiometers (Interacoustics AS Company, Denmark) calibrated previously were used to test binaural air and bone conduction threshold audiometry at 0.5, 1, 2, 3, 4, 6 kHz. The surrounding should be quiet and the noise background value <25 dB(A). The results of the tests were collected by age and gender.

### Calculation of CNE

**Fig. 1:**
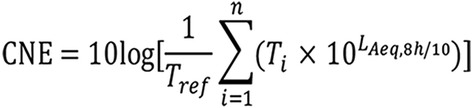
The interpretation of CNE equation. Where Tref is equal to 1; n is the total number of different positions for the workers exposed to noise; i is the number of different posts; T is the time at different positions; L_Aeq, 8h_ is the equivalent continuous sound level of 8 h for different jobs

The equivalent continuous sound level (A) (L_Aeq, 8h_) was measured using Noisepro multi-functional individual noise dosimeters (NoisePro series, Quest Technologies, USA) which were adjuested by type of QC-10 Sound calibrators before measurement. The noise dosimeters were set as weight of A, S (slow), the value of L_Aeq, 8h_ and then the CNE for every subjects was calculated based on the Fig. 1 (the Footnotes of Fig. 1 at the end of the article) [6].

### DNA extraction

Peripheral blood (≥2 ml) was collected in the EDTA anticoagulant tube and stored at minus 80 °C. The DNA of the peripheral blood was extracted using a 2 ml blood genomic DNA extraction kit (Shanghai Laifeng Biotech, Shanghai, Chnia). The concentration and purity of DNA were measured using the NanoPhotometer P360 ultramicro spectrophotometer (Shanghai Boyibio Biotech, Shanghai, China). The prerequisites for subsequent DNA genotyping were that the A260/A280 value was between 1.8 and 2.0 and the concentration was >50 ng/μL.

### SNP selection and genotyping

SNPs were selected according to the 1000 Genomes Project resources (http://www.internationalgenome.org/) and relative literatures and the inclusion criteria were as follows:

1. sites of SNPs laid in the area of *GRM7* with the minor allele frequency (MAF) > 0.10 in the Chinese Han population;
2. the linkage disequilibrium (LD) method of SNPs with the pairwise r^2^ > 0.80.

Five SNPs in *GRM7* were selected in this study.

In this study, the SNPs were genetyped using the SNPscan multiplex SNP genotyping kits (Genesky Biopharm Technology, Shanghai, China). AB13730XL DNA analyser was used to detect the sequence and GeneMapper 4.1 software (Applied Biosystems, USA) was utilized to analyze the genotype of SNP loci.

### Statistical analysis

Data was analyzed using SPSS21.0 software. The continuous variables were implemented tests of normality and all of them were not in accordance with normal distribution, hence they were expressed by the median (range) and the differences between groups were analysed by Wilcoxon rank sum test. All of the classification variables were expressed by frequencies (percentile) and the comparisons between groups were analysed by pearson chi-square test.The control groups of SNPs were tested whether they were in line with Hardy-Weinberg equilibrium using Pearson’s χ^2^ test. Four genetic models (additive model, dominant model, recessive, codominant model) were established and a conditional logistic regression was implemented to analyse the relationship between the polymorphisms of *GRM7* and the susceptibility to NIHL. The interactions of genetic and environmental factors were also considered in logistic regression analysis by the option of multiplying interaction effect, and if the interactions were significant, then the stratification would be carried out to analyze the main effects. The generalised multiple dimensionality reduction software V.0.9 (GMDR V.0.9) was applied to find the interaction among SNPs. The possible confounders, such as CNE, smoking, drinking, hypertension, were adjusted during the statistical analysis. The test level was α = 0.05 therefore it was statistically significant if *p* < 0.05. All the testing hypotheses were two-tailed. Bonferroni correction was used in the multiple comparing by pairs.

## Results

### Evaluation of matching effect in case and control groups

In this study, a total of 876 people were involved, in which there were 292 cases and 584 controls, aging from 20.75 to 59.25 years old. As showed in Table 1 (after references), the matching effect was evaluated in the case and control groups through comparing the basic information distribution between them.

**Table 1:** Basic Information Distribution in Case and Control Groups

Variables	Case (*n* = 292)	Control (*n* = 584)	Statistics	*p*
Age, year				
20~30	45 (15.4%)	104 (17.8%)		
30~40	79 (27.1%)	153 (26.2%)		
40~50	134 (45.9%)	270 (46.2%)		
50~60	34 (11.6%)	57(9.8%)	1.381^‡^	0.710
Noise exposure duration, year	18.860 (8.500, 27.750)	18.509 (8.167, 26.917)	−0.692^§^	0.489
CNE, dB(A)^*^	97.844 (94.686, 101.522)	97.767 (94.854, 101.195)	−0.153^§^	0.878
HTL, dB(A) ^†^	50.980 (44.042, 55.833)	18.293 (12.500, 24.000)	−24.153^§^	<0.001
Height, cm	170.366 (167.000, 174.750)	169.993 (166.000, 174.000)	−1.004^§^	0.315
Gender				
Male	281 (96.2%)	560 (95.9%)		
Female	11 (3.8%)	24 (4.1%)	0.060^‡^	0.807
level of environmental noise exposure, dB(A)				
≤ 85	121 (41.4%)	254 (43.5%)		
> 85	171 (58.6%)	330 (56.5%)	0.336^‡^	0.562
Tinnitus				
Yes	196 (67.4%)	316 (54.2%)		
No	95 (32.6%)	267 (45.8%)	12.837^‡^	<0.001
Smoking				
Yes	181 (62.0%)	341 (58.4%)		
No	109 (38.0%)	243 (41.6%)	1.045^‡^	0.307
Drinking				
Yes	203 (69.5%)	399 (68.3%)		
No	89 (30.5%)	185 (31.7%)	0.130^‡^	0.718
Hypertension				
Yes	112 (38.4%)	242 (41.4%)		
No	180 (61.6%)	342 (58.6%)	0.768^‡^	0.381

Evaluation of the matching effects in the case and control groups by comparing the basic information distribution between them

^*^CNE: cumulative noise exposure

^†^HTL: the binaural average hearing threshold level in high frequencies

^‡^Pearson chi-square test

^§^Wilcoxon rank sum test

**Fig. 2:**
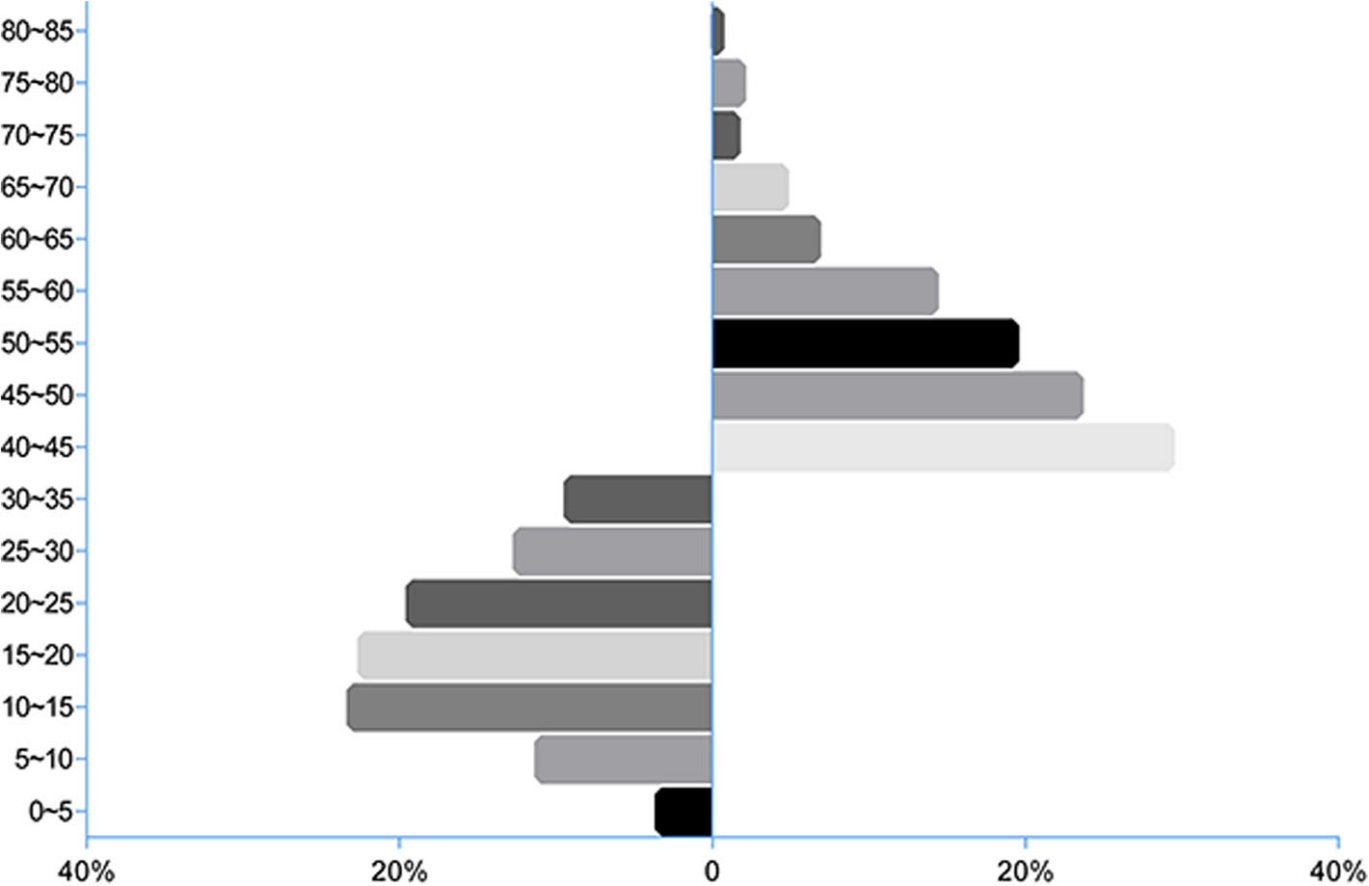
The proportional distribution of the binaural average hearing threshold levels (HTLs) at 3 kHz,4 kHz,6 kHz in case and control groups. HTL is grouped by 5 dB(A) in both case and control groups. The control group ranges from 0 to 35 dB(A) and the case group is in the range of 40 to 85 dB(A)

By test of normality, the whole continuous variables did not conform to the normal distribution, so nonparametric test was used. The classification variables were analysed by pearson χ^2^ test. The binaural average hearing threshold level (HTL) (3 kHz,4 kHz,6 kHz) in the case group was higher than that of the control group, which conformed to the design needs of our research (*p* < 0.001). What’s more, the proportion of tinnitus in the case group was higher than that of the control group with *p* < 0.001. Other comparisons in the case and control groups, including the general demographic characteristics (age, sex, height, noise exposure duration), individual factors (smoking, drinking), disease history (hypertension), and the observing indicators (CNE, level of environmental noise exposure) were of no significant difference (*p* > 0.05). The proportional distribution of HTL in case and control groups was showed in Fig. 2 (the Footnotes of Fig. 2 at the end of the article).

### Assessment of genotype effects on NIHL

#### Hardy-Weinberg equilibrium for control group

Table 2 (after references) showed that *p* values in the control group of the selected SNPs were all above 0.05, which demonstrated that all SNPs in control group were in Hardy-Weinberg equilibrium (HWE) and the selected population of the control group was representative.

**Table 2:** Basic Information of the Selected SNPs

SNP	Chromosomal position	MAF^*^	Allele*		χ^2b^	*p* ^†^
			Ancestral allele	Mutant allele		
rs11920109	Chr3:7,212,686	0.4016	*T* = 0.4272	C = 0.5728	2.4271	0.2178
rs1485175	Chr3:7,620,789	0.4744	*T* = 0.5437	C = 0.4563	1.3677	0.2589
rs9819783	Chr3:7,208,213	0.3848	T = 0.4272	C = 0.5728	1.5317	0.3961
rs9826579	Chr3:7,782,371	0.3317	C = 0.1408	*T* = 0.8592	0.0185	0.9603
rs9877154	Chr3:7,159,406	0.4187	*T* = 0.3932	C = 0.6068	0.1975	0.9045

Hardy-Weinberg equilibrium test of all selected SNPs in the control group

^b^Pearson chi-square test is used to test whether the SNPs in the control group is in line with Hardy-Weinberg equilibrium

*The data comes from NCBI dbSNP and 1000 Genomes Browser (CHB)

^†^Hardy-Weinberg Equilibrium Test of the control group by Pearson’s χ^2^

#### Construction of genetic models and effect analysis

Four genetic models were constructed for every SNP in this study: the additive model (wtwt, wtmt, mtmt), dominant model [(mtmt + wtmt) vs wtwt], recessive model [mtmt vs (wtwt + wtmt)], codominant model (wtwt vs wtmt vs mtmt) (wt: wild type, mt: mutant type) and then conditional logistic regression analysis was conducted to evaluate the effect of the genetic models. Results in Table 3 (after references) indicated that in additive model CC genotype of rs1485175 had a protective effect to the risk of developing NIHL with an adjusted OR of 0.564 (*p*/*p*_bon_ = 0.008/0.002, 95% CI: 0.370–0.860). But statistical association with NIHL was not found in TC genotype. In dominant model of rs1485175, statistical significant difference with a decreased risk was found (*p*/*p*_bon=_ 0.050/0.010, OR = 0.737, 95% CI: 0.554–1.000) and the associations in same direction were also detected in the recessive/codominant model and the allele C/T with the *p*/*p*_bon_ value of 0.018/0.004 (OR = 0.636, 95%CI: 0.437–0.925), 0.009/0.002 (OR = 0.761, 95%CI: 0.620–0.934) and 0.017/0.003 (OR = 0.800, 95%CI: 0.666–0.962), respectively. No significant associations were discovered in any other four SNPs between the case and control groups.

**Table 3:** Correlation of Genetic Models with Risk of Developing NIHL

SNP	Genotype	Case		Control		OR (95%CI)*	*p*/*p*_bon_^†^
		n	%	n	%		
rs11920109	TT	40	13.7	92	15.8	1	0.738/0.148
	TC	153	52.4	297	50.9	1.182 (0.774, 1.806)	0.439/0.088
	CC	99	33.9	194	33.3	1.141 (0.724, 1.800)	0.531/0.106
	CC + TC	252	86.3	491	84.2	1.155 (0.736, 1.814)	0.443/0.089
	TT + TC	193	66.1	389	66.7	1	
	CC	99	33.9	194	33.3	1.016 (0.745, 1.386)	0.920/0.184
	TT/TC/CC					1.055 (0.850,1.309)	0.627/0.125
	Allele C/T					1.039 (0.866, 1.246)	0.682/0.136
rs1485175	TT	103	35.5	169	29.1	1	0.029/0.006
	TC	139	47.9	276	47.5	0.820 (0.593, 1.132)	0.227/0.045
	CC	48	16.6	136	23.4	0.564(0.370, 0.860)	0.008/0.002
	CC + TC	187	64.5	412	70.9	0.737 (0.544, 1.000)	0.050/0.010
	TT + TC	242	83.4	445	76.6	1	
	CC	48	16.6	136	23.4	0.636 (0.437, 0.925)	0.018/0.004
	TT/TC/CC					0.761 (0.620, 0.934)	0.009/0.002
	Allele C/T					0.800 (0.666, 0.962)	0.017/0.003
rs9819783	TT	38	13.1	86	14.8	1	0.785/0.157
	TC	148	50.9	288	49.5	1.163 (0.753, 1.797)	0.496/0.099
	CC	105	36.1	208	35.7	1.150 (0.728, 1.816)	0.550/0.110
	CC + TC	253	86.9	496	85.2	1.158 (0.765, 1.752)	0.489/0.098
	TT + TC	186	63.9	374	64.3	1	
	CC	105	36.1	208	35.7	1.024 (0.751, 1.396)	0.881/0.176
	TT/TC/CC					1.054 (0.848, 1.309)	0.637/0.127
	Allele C/T					1.038 (0.864, 1.248)	0.688/0.138
rs9826579	CC	8	2.7	16	2.7	1	0.718/0.144
	CT	73	25.1	162	27.8	0.938 (0.369, 2.386)	0.893/0.179
	TT	210	72.2	404	69.4	1.072 (0.438, 2.622)	0.879/0.176
	TT + CT	283	97.3	566	97.3	1.049 (0430, 2.559)	0.917/0.183
	CC + CT	81	27.8	178	30.6	1	
	TT	210	72.2	404	69.4	1.136 (0.833, 1.549)	0.422/0.084
	CC/CT/TT					1.106 (0.844, 1.451)	0.465/0.093
	Allele T/C					1.086 (0.849, 1.390)	0.510/0.102
rs9877154	TT	41	14.0	86	14.7	1	0.712/0.142
	TC	147	50.3	278	47.6	1.125 (0.737, 1.719)	0.585/0.117
	CC	104	35.6	220	37.7	0.995 (0.636, 1.556)	0.983/0.197
	CC + TC	251	86.0	498	85.3	1.071 (0.715, 1.604)	0.740/0.148
	TT + TC	188	64.4	364	62.3	1	
	CC	104	35.6	220	37.7	0.909 (0.670, 1.233)	0.539/0.108
	TT/TC/CC					0.973 (0.787, 1.202)	0.799/0.160
	Allele C/T					0.980 (0.815, 1.177)	0.825/0.165

Effects of genetic models evaluated by conditional logistic regression

*CNE, height, smoking, drinking, and hypertension are adjusted; CI: Confidence interval

^†^Bonferroni correction is used to adjust *p* values by means of 0.05 / 5 (5 selected SNPs) to get *p*_bon_ values of 0.01, which means that it is significant in statistics if *p*_bon_ < 0.01

#### Interaction Analysis between Genes and Environment

The interaction between genes and the environment (CNE) was analyzed by the option of multiplying interaction effect in logistic regression analysis. The analysis found that rs1485175 and CNE had a significant interaction in statistics with p = 0.007 and OR = 0.997 (95% CI: 0.995–0.999). Then, the main genotype effect in each layer (< 97 dB (A) and > 97 dB(A)) was figured out. Significant differences were found in TT and CC with the CNE > 97 dB (A). The specific results of the analysis could be found in Table 4.

**Table 4:** Relationship between rs1485175 and NIHL layered by CNE

Environmental factor	Genotype	Case		Control		OR (95% CI) ^*^	*p*/*p*_bon_^†^
		n	%	n	%		
CNE, dB (A)							
< 97	TT	44	30.8	81	29.5	1	0.846/0.423
	TC	74	51.7	138	50.2	1.023 (0.700, 1.496)	0.906/0.453
	CC	25	17.5	56	20.4	0.895 (0.546, 1.469)	0.662/0.331
> 97	TT	61	40.9	91	29.4	1	0.047/0.024
	TC	65	43.6	138	44.7	0.784 (0.550, 1.118)	0.179/0.090
	CC	23	15.4	80	25.9	0.550 (0.340, 0.891)	0.014/0.007

The interaction between genes and the environment (CNE) analyzed by layering

^*^Height, smoking, drinking, and hypertension are adjusted

^†^The statistically significant *p* values are adjusted by Bonferroni correction through 0.05/2 (2 groups layered by CNE) and it is statistically significant if *p*_bon_ < 0.025

#### Analysis of interaction effects among SNPs

**Fig. 3:**
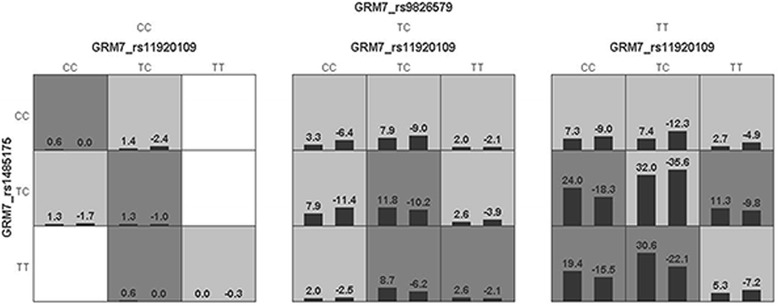
Best model gained by the analysis of GMDR. The implications of bars and background color in each multifactor cell are as follows. The left bars represent the sum of scores in case and the right represent the control. High risk cells are expressed by black shadow if the ratio of the number of cases to the number of controls exceeds the preset value T, as low risk cells by light shadow if not more than the threshold and empty cells by no shadow which means no cases and controls. The multifactor cells labeled as “high risk” or “low risk” are then used to assess the classification and predication accuracy, thus identifying the best model in the subsequent steps

The generalized multifactor dimensionality reduction (GMDR) v0.9 was applied in this research to detect the interaction of the 5 selected SNPs in *GRM7*. Covariates, CNE, height, smoking, drinking, hypertension, were adjusted in the analysis by loading phenotype data. Table 5 (after references) provided the best model, testing balanced accuracy, cross-validation (CV) consistency, and *p* values by sign test. In all of the models the combination of rs1920109, rs1485175, rs9826579 formed the best model with a statistically significant p value of 0.0547, the maximum testing balanced accuracy of 53.55% and the biggest CV consistency (9/10). Permutation test appeared that the best model made of rs1920109, rs1485175, rs9826579 statistically significant (*p* = 0.037). Figure 3 (the Footnotes of Fig. 3 at the end of the article) showed the best model constituted by rs11920109, rs1485175, rs9826579.

**Table 5:** Results of the best model identified by GMDR

Best model^*^	Testing balanced accuracy (%)	CV consistency ^†^	*p* ^‡^	*p* ^§^
rs1485175	52.62	10/10	0.6230	0.135
rs1485175, rs9877154	50.89	4/10	0.3770	0.141
rs11920109, rs1485175, rs9826579	53.55	9/10	0.0547	0.037
rs11920109, rs1485175, rs9819783, rs9826579	51.09	7/10	0.6230	0.126
rs11920109, rs1485175, rs9819783, rs9826579, rs9877154	50.59	10/10	0.6230	0.403

The analysis of the interaction of the 5 selected SNPs in *GRM7*

^*^CNE, height, smoking, drinking, hypertension are adjusted

^†^CV consistency means cross-validation consistency

^‡^Based on sign test

^§^Based on permutation test

## Discussion

The whole genome association study has found that the T-allele of rs11928865 in *GRM7* is associated with age-related hearing loss (ARHI) in European group, which is also confirmed in an elderly male Han Chinese population and rs779706 and rs779701, the SNPs within *GRM7*, are significant in the Finnish group [21, 30]. Although the polymorphisms of *GRM7* are an important factor affecting ARHI, genetic polymorphisms are also common in general population and they should also been involved in the study of NIHL.

Our results discovered for the first time that the mutant allele C in rs1485175 of the *GMR7* had significant associations with NIHL among the additive, dominant, recessive, codominant models and the allele C/T. The result suggests that CC genotype might act as a protective factor for the glutamate toxicity caused by high intensity noise exposure. That’s to say in the same harmful noisy environment, individuals with non-CC genotype in rs1485175 of *GRM7* are more susceptible than those who have CC genotype. But in our study we have not found the statistical correlation between the rs11928865 of *GRM7* and the susceptibility to NIHL.

Many researches have demonstrated that NIHL is a disease resulted from the interaction of gene and environment. Therefore the interaction between rs1485175 and environmental factors and the interaction of rs1485175 with the other selected SNPs of *GRM7* were analyzed. The results of the gene-by-environment interaction analysis suggest that CNE at higher level (> 97 dB(A)) might interact with rs1485175 with the *p* value of 0.014 and OR of 0.550 (95% CI: 0.340–0.891). The risk of developing NIHL reduces by 0.447 after stratification according to CNE, which suggests that CNE may be a risk factor for NIHL and workers are more susceptible to noise at the higher level of CNE [6]. The gene-by-gene interaction data obtained through the software of GMDR showed that the best model made up of rs11920109, rs1485175, rs9826579, suggesting that the main effect of the *GRM7* gene on susceptibility to NIHL we have found is the combination of these three genes; that is, the influence of rs1485175 on NIHL is dependent on the genotypes of the other two SNPs in *GRM7*.

High glutamate concentration can lead to programmed death of the spiral ganglion neurites and cells, which is also verified by incubating spiral ganglion explants with a caspase-3 inhibitor (an inhibitor of apoptosis) [27]. Therefore, the negative feedback regulation of glutamate is important in maintaining the normal transmission of the sound signal in the ear. The mGluR7, Group III mGlu receptors, is principally situated in the presynaptic membrane and negatively regulates the level of glutamate. The activation of mGluR7 can hinder glutamate releasing when exposed in high intensity noise level, which lowers the excitability of target cells and neurons, thus playing a protective role in glutamate excitotoxicity [31].

It is possible that the T-allele mutation to C-allele in rs1485175 may take part in this process. In the normal inner hair cells and neurons, the mutant allele C in rs1485175 may increase the number of mGluR7 or enhance its sensitivity to glutamate in the presynaptic membrane, and then to accelerate the glutamate uptake or reduce the release of glutamate when exposed to high intense noise, thus avoiding the glutamate excitotoxicity. That may be the reason why individuals with CC genotype of rs1485175 in *GRM7* are less susceptible to noise than non-CC genotype. CNE [6], height [32], smoking [33], drinking [34, 35], and hypertension [35], which are all hazardous factors for NIHL, were adjusted in the process of analyzing.

Advantages for this study are as follows. Firstly, a nested case-control study used in our study can better overcome the selection bias and recall bias, as well as reducing costs. Secondly, more accurate measurements than previous studies are used, including the intensity of noise exposure, the diagnosis of NIHL and the principle of double blind and so on. Some limitations of this study should also be known. It has been known that exposing to noise coming from living environment and individual activities for a long time can also cause hearing impairment [36], but the effect is smaller and less hazardous compared with NIHL due to noise exposure in the working place [37]. Namely, the results of our study are authentic.

## Conclusions

In summary, we find for the first time that the mutant allele C of rs1485175 in *GRM7* may reduce the susceptibility of individuals to NIHL in Chinese Han population. At the same time, we also note that the gene-by-environmental and gene-by-gene interactions may affect the protective effect of mutant allele C in rs1485175 of *GRM7*. This finding, once verified by large studies, will have important implications in the prevention of NIHL in susceptible occupational population.

## Additional file

Additional file 1: The supplementary material was designed for this study. The data in the questionnaire titled “the Questionnaire of Occupational Health” and they contained the basic demographic information of the workers, the information of smoking and drinking, occupational history, the history of past disease and drug use, family history of deafness, work-related injuries and other information related to the occupational health. (DOCX 45 kb)

## Abbreviations

ARHI: Age-related hearing loss
CNE: Cumulative noise exposure
GMDR: Generalized multiple dimensionality reduction
*GRM7*: Metabolic glutamate receptor7 gene
HTLs: Hearing threshold levels
HWE: Hardy-Weinberg equilibrium
LD: Linkage disequilibrium
MAF: Minor allele frequency
mGluR7: Metabotropic glutamate receptor 7
mt: Mutant type
NIHL: Noise-induced hearing loss
SNPs: Single nucleotide polymorphisms
wt: Wild type

## References

[1] Basner M, Babisch W, Davis A, Brink M, Clark C, Janssen S, Stansfeld S (2014). Auditory and non-auditory effects of noise on health. Lancet.

[2] Corvalan. aPUJLSTC: Chapter 21 Selected occupational risk factors. comparative quantification of Health risks. global and regional Burden of disease attributable to selected major risk factors. 2004, Vol 2(1651–1802.

[3] WHO: Protecting workers' health. 2014. [http://www.who.int/mediacentre/factsheets/fs389/en/]. Accessed 25 June 2017.

[4] Leigh JP (2011). Hearing impairment among noise-exposed workers - United States, 2003-2012. Milbank Q.

[5] NIOSH: NIOSE AND HEARING LOSS PREVENTION. [https://www.cdc.gov/niosh/topics/noise/prevention.html]. 2016. Assessed 26/06/2017.

[6] SF Y, Chen G, Jiao J, GZ G, Zhang HL, Wang XM, Zhou WH, Wu H, Li YH, Zheng YX (2017). A cohort study on occupational noise induced hearing loss in workers at an iron and steel plant. Chinese Journal of Preventive Medicine.

[7] Konings A, Van Laer L, Van Camp G (2009). Genetic studies on noise-induced hearing loss: a review. Ear Hear.

[8] Delmaghani S, Defourny J, Aghaie A, Beurg M, Dulon D, Thelen N, Perfettini I, Zelles T, Aller M, Meyer A (2015). Hypervulnerability to sound exposure through impaired adaptive proliferation of peroxisomes. Cell.

[9] Kozel PJ, Davis RR, Krieg EF, Shull GE, Erway LC (2002). Deficiency in plasma membrane calcium ATPase isoform 2 increases susceptibility to noise-induced hearing loss in mice. Hear Res.

[10] Housley GD, Morton-Jones R, Vlajkovic SM, Telang RS, Paramananthasivam V, Tadros SF, Wong AC, Froud KE, Cederholm JM, Sivakumaran Y (2013). ATP-gated ion channels mediate adaptation to elevated sound levels. Proc Natl Acad Sci U S A.

[11] Holme RH, Steel KP (2004). Progressive hearing loss and increased susceptibility to noise-induced hearing loss in mice carrying a Cdh23 but not a Myo7a mutation. J Assoc Res Otolaryngol.

[12] Ohlemiller KK, McFadden SL, Ding DL, Flood DG, Reaume AG, Hoffman EK, Scott RW, Wright JS, Putcha GV, Salvi RJ (1999). Targeted deletion of the cytosolic cu/Zn-superoxide dismutase gene (Sod1) increases susceptibility to noise-induced hearing loss. Audiol Neurootol.

[13] Ohlemiller KK, McFadden SL, Ding D-L, Lear PM, Ho Y-S (2000). Targeted mutation of the gene for cellular glutathione peroxidase (Gpx1) increases noise-induced hearing loss in mice. J Assoc Res Otolaryngol.

[14] Van Laer L, Carlsson PI, Ottschytsch N, Bondeson ML, Konings A, Vandevelde A, Dieltjens N, Fransen E, Snyders D, Borg E (2006). The contribution of genes involved in potassium-recycling in the inner ear to noise-induced hearing loss. Hum Mutat.

[15] Pawelczyk M, Van Laer L, Fransen E, Rajkowska E, Konings A, Carlsson PI, Borg E, Van Camp G, Sliwinska-Kowalska M (2009). Analysis of gene polymorphisms associated with K ion circulation in the inner ear of patients susceptible and resistant to noise-induced hearing loss. Ann Hum Genet.

[16] Konings A, Van Laer L, Wiktorek-Smagur A, Rajkowska E, Pawelczyk M, Carlsson PI, Bondeson ML, Dudarewicz A, Vandevelde A, Fransen E (2009). Candidate gene association study for noise-induced hearing loss in two independent noise-exposed populations. Ann Hum Genet.

[17] Yang M, Tan H, Yang Q, Wang F, Yao H, Wei Q, Tanguay RM, Wu T (2006). Association of hsp70 polymorphisms with risk of noise-induced hearing loss in Chinese automobile workers. Cell Stress Chaperones.

[18] Li YH, Yu SF, Gu GZ, Chen GS, Zheng YX, Jiao J, Zhou WH, Wu H, Zhang ZR, Zhang HL (2017). Polymorphisms of heat shock protein 70 genes (HSPA1A, HSPA1B and HSPA1L) and susceptibility of noise-induced hearing loss in a Chinese population: a case-control study. PLoS One.

[19] Yang QY, XR X, Jiao J, Zheng YX, He LH, SF Y, GZ G, Chen GS, Zhou WH, Wu H (2016). Genetic variation in EYA4 on the risk of noise-induced hearing loss in Chinese steelworks firm sample. Occup Environ Med.

[20] XR X, Yang QY, Jiao J, He LH, SF Y, JingWang J, GZ G, Chen GS, Zhou WH, Wu H (2016). Genetic variation in POU4F3 and GRHL2 associated with noise-induced hearing loss in Chinese population: a case-control study. Int J Environ Res Public Health.

[21] Friedman RA, Laer LV, Huentelman MJ, Sheth SS, Eyken EV, Corneveaux JJ, Tembe WD, Halperin RF, Thorburn AQ, Thys S (2009). GRM7 variants confer susceptibility to age-related hearing impairment. Hum Mol Genet.

[22] Newman DL, Fisher LM, Ohmen J, Parody R, Fong CT, Frisina ST, Mapes F, Eddins DA, Robert Frisina D, Frisina RD, Friedman RA (2012). GRM7 variants associated with age-related hearing loss based on auditory perception. Hear Res.

[23] Van Laer L, Huyghe JR, Hannula S, Van Eyken E, Stephan DA, Maki-Torkko E, Aikio P, Fransen E, Lysholm-Bernacchi A, Sorri M (2010). A genome-wide association study for age-related hearing impairment in the Saami. Eur J Hum Genet.

[24] Puel JL (1995). Chemical synaptic transmission in the cochlea. Prog Neurobiol.

[25] Hakuba N, Koga K, Gyo K, Usami SI, Tanaka K (2000). Exacerbation of noise-induced hearing loss in mice lacking the glutamate transporter GLAST. J Neurosci.

[26] Pujol R, J-L Puel, D'aldin CG, Eyblin M. Pathophysiology of the glutamatergic synapses in the cochlea. Acta Otolaryngol. 2009;113(3):330–4. 10.3109/00016489309135819 8100108

[27] Steinbach S, Lutz J (2007). Glutamate induces apoptosis in cultured spiral ganglion explants. Biochem Biophys Res Commun.

[28] Eybalin M, Norenberg MD, Renard N (1996). Glutamine synthetase and glutamate metabolism in the guinea pig cochlea. Hear Res.

[29] Henderson D, Bielefeld EC, Harris KC, BH H (2006). The role of oxidative stress in noise-induced hearing loss. Ear Hear.

[30] Luo HJ, Yang T, Jin XJ, Pang XH, Li JP, Chai YC, Li L, Zhang Y, Zhang LP, Zhang ZH (2013). Association of GRM7 variants with different phenotype patterns of age-related hearing impairment in an elderly male Han Chinese population. PLoS One.

[31] Williams CJ, Dexter DT (2014). Neuroprotective and symptomatic effects of targeting group III mGlu receptors in neurodegenerative disease. J Neurochem.

[32] Burr H, Lund SP, Bügel Sperling B, Kristensen TS, Poulsen OM (2009). Smoking and height as risk factors for prevalence and 5-year incidence of hearing loss. A questionnaire-based follow-up study of employees in Denmark aged 18–59 years exposed and unexposed to noise. Int J Audiol.

[33] Tao L, Davis R, Heyer N, Yang Q, Qiu W, Zhu L, Li N, Zhang H, Zeng L, Zhao Y (2013). Effect of cigarette smoking on noise-induced hearing loss in workers exposed to occupational noise in China. Noise Health.

[34] Upile T, Sipaul F, Jerjes W, Singh S, Nouraei SA, El Maaytah M, Andrews P, Graham J, Hopper C, Wright A: The acute effects of alcohol on auditory thresholds. BMC Ear Nose Throat Disord 2007, **7**(4. 10.1186/1472-6815-7-4 PMC2031886 17877829

[35] Przewozny T, Gojska-Grymajlo A, Kwarciany M, Gasecki D, Narkiewicz K (2015). Hypertension and cochlear hearing loss. Blood Press.

[36] Hammer MS, Swinburn TK, Neitzel RL (2014). Environmental noise pollution in the United States: developing an effective public health response. Environ Health Perspect.

[37] Lie A, Skogstad M, AH, Johannessen, Tynes T, Mehlum IS, Nordby K-C, Engdahl B, Tambs K: Occupational noise exposure and hearing: a systematic review. Int Arch Occup Environ Health 201689)**:**351–372. 10.1007/s00420-015-1083-5 PMC4786595 26249711

