# Supplementary material for: Effect of GRM7 polymorphisms on the development of noise-induced hearing loss in Chinese Han workers: a nested case-control study
Source: BMC Med Genet. 2018 Jan 5;19:4. doi: 10.1186/s12881-017-0515-3 (PMC5755024; doi:10.1186/s12881-017-0515-3)
Supplement: Supplementary file 1 — The supplementary material was designed for this study. The data in the questionnaire titled “the Questionnaire of Occupational Health” and they contained the basic demographic information of the workers, the information of smoking and drinking, occupational history, the history of past disease and drug use, family history of deafness, work-related injuries and other information related to the occupational health. (DOCX 45 kb) [file 12881_2017_515_MOESM1_ESM.docx]

**Questionnaire of Occupational Health**

**A. Basic information (Please tick "√" on the number box before the answer of your choice)**

A1 Kind of work:

Manager Worker

A2 Gender:

Male Female

A3 Date of birth:

A4 Nationality:

A5 Marital status:

Married Unmarried Widowed Divorced

A6 Educational level:

Have never been to school Primary school Middle school High school Vocational school Junior college and above

A7 Current personal monthly income:

1000 to 2000 yuan 2000 to 3000 yuan 3000 to 4000 yuan 4000 to 5000 yuan above 5000 yuan

A8 Current income per capita:

≤ 300 yuan 300 to 500 yuan 500 to 800 yuan 800 to 1200 yuan 1200 to 2000 yuan above 2000

A9 Initial diagnosis time of noise-induced hearing loss: ____;

The first confirmed unit: ____;

The initial diagnosis level: ____.

**B. The history of smoking (Remark: If you have given up smoking, please fill in according to the situation before quitting.)**

B1 Smoking status:

Smoking No smoking Have quit smoking Years for quitting smoking: ____

B2 Smoking amount:

5 cigarettes a day 5-10 cigarettes a day 10-20 cigarettes a day >20 cigarettes a day

B3 Age of beginning smoking: ____

B4 Total years of smoking: ____

**C. The history of drinking (Remark:** **If you have given up smoking, please fill in according to the situation before quitting.)**

C1 Drinking situation:

Drinking No drinking Have quit drinking Years for quitting drinking: ____

C2 Age of beginning drinking: ____

C3 Total years of drinking: ____

**Questionnaire 1 of drinking times**

|  | **C4**  **≤ 2 times/month** | **C5**  **2-4 times/month** | **C6**  **2-4 times/week** | **C7**  **≥5 times/week** |
| --- | --- | --- | --- | --- |
| Beer |  |  |  |  |
| Wine (red wine) |  |  |  |  |
| White spirit |  |  |  |  |

**D. Field work**

D1 You work an average of _____ hours per day in noisy workplaces.

D2 Are there any other occupational hazards in your workplace besides noise?

Yes A. vibration B. dust C. heat D. others: _____

No

**E. The history of occupation**

**Questionnaire 8 of worker’s occupational history**

| **E1**  **Time of beginning** | **E2**  **Time of ending** | **E3**  **Work unit** | **E4**  **Workshop/ Departments** | **E5**  **Workshop section/ work team** | **E6**  **Type of work** | **E7**  **Occupational hazards** |
| --- | --- | --- | --- | --- | --- | --- |
|  |  |  |  |  |  |  |
|  |  |  |  |  |  |  |
|  |  |  |  |  |  |  |

**F. The history of past disease**

**Questionnaire 2 of past disease history**

(Remark: If the disease you have suffered was listed in questionnaire 2, please write “1” after this disease; otherwise “0”.)

| **Names of diseases** | **1/0** | **Names of disease** | **1/0** |
| --- | --- | --- | --- |
| F1 History of head trauma |  | F20 Hypertension |  |
| F2 Ear barotrauma |  | F21 Diabetes |  |
| F3 Blast exposure hearing damage |  | F22 Hyperlipoidemia |  |
| F4 Congenital deafness |  | F23 Atherosclerosis |  |
| F5 Sudden deafness |  | F24 Hypothyroidism |  |
| F6 Otitis media |  | F25 Polycythemia |  |
| F7 Ear shingles |  | F26 Sickle cell anemia |  |
| F8 Meniere's syndrome |  | F27 Multiple sclerosis |  |
| F9 Autoimmune sensorineural deafness |  | F28 Multiple tuberous arteritis |  |
| F10 Cholesteatoma |  | F29 Chronic nephritis and renal failure |  |
| F11 Systemic lupus erythematosus |  | F30 Leukemia |  |
| F12 Endemic cretinism |  | F31 Influenza |  |
| F13 Measles |  | F32 Mumps |  |
| F14 Rubella |  | F33 Scarlatina |  |
| F15 Chickenpox |  | F34 Malaria |  |
| F16 Syphilis |  | F35 Epidemic encephalitis B |  |
| F17 Epidemic cerebrospinal meningitis |  | F36 AIDS |  |
| F18 Typhoid fever |  | F37 Alcoholism |  |
| F19 Typhus |  | F38 Carbon monoxide poisoning |  |

Other diseases: ________________________________________________________
(Remark: If the diseases you have suffered not included in questionnaire 2, please list them in detail. )

**G. The history of past drug use**

**Questionnaire 3 of past drug use history**

(Remark: If you have ever taken the drug listed in questionnaire 3, please write “1” after the drug; otherwise “0”.)

| G1 **Aminoglycoside & vancomycin antibiotics**  (such as: gentamicin, kanamycin, neomycin, streptomycin, amikacin, netilmicin; vancomycin) |  | G5 **Sedative hypnotic drugs, Anticonvulsant drugs**  (such as: Diazepam (valium), phenobarbital (lumial), promethazine (Phenergan), chlorpromazine, estazolam (isazolam), amitriptyline, clozapine, perphenazine, sulpiride, et al.) |  |
| --- | --- | --- | --- |
| G2 **Antineoplastic**  (such as: furosemidum, ethacrynic acid, BuMetanide, et al.) |  | G6 **Heavy metal preparations**  (such as: arsenical (realgar, bezoar antidotal tablet, maren boluses, et al), Lead agent (minium), mercurial (cinnabar) and so on.) |  |
| G3 **Antineoplastic drugs**  (such as: cis platinum diamminedicbloride (cDDP), vincristine, nitrogen mustard and so on.) |  | G7 Contraceptive  (such as: Mifepristone tablets (fenel), Levonogesterone tablet (yuting), et al.) |  |
| G4 **Antipyretic analgesics**  (such as: Aspirin, naproxen, ibuprofen, paracetamol, and so on.) |  | G8 **Antimalarial drugs**  (such as: quinine, hydroxychloroquine, euquinine, arteether, et al.) |  |

Other medications: ____________________________________________________

**H. Family history of deafness: Yes No (If the answer is yes, please fill the following questionnaire 4.)**

**Questionnaire 4 of the workers’ family deafness history**

| **H1 The relationship with the worker** | **H2 Confirmed time** |
| --- | --- |
|  |  |
|  |  |

**I. Health effects of noise and noise protection**

I1 How do you feel about your hearing? good ordinary bad very bad

I2 Do you have any symptoms of tinnitus? (Especially after work) Yes No

I3 In a noisy environment, do you think listening to music with headphones can reduce the health effects of noise? Yes No

I4 How do you think noise affects human health? By causing hearing impairment By affecting the blood pressure By increasing the incidence of cardiovascular disease By affecting the digestive function By causing headache, dizziness, insomnia and so on. By affecting mood

I5 Will the impact of noise on health reduced after accustomed to the noise environment? Yes No

I6 Have you worn the earplugs and other protective equipment during the working time? Yes Basically wear Basically do not wear Do not wear

I7 Do you think the earplugs that the unit currently dispensed can effectively reduce noise? Very effective A little effective No significant effect Totally ineffective

**J. Physical exercise**

J1 Do you often participate in physical exercise? Yes No

J2 Your regular physical activities: fast walking Play football Play basketball Play badminton Play table tennis Others: _________

**K. Work-related injury Yes No**

**Questionnaire 5 of employee's work accident situation**

| **K1 Time of occurrence** | **K2 Location of accident** | **K3 Reasons of work accident** | **K4 Degree of disability** | **K5**  **Prognosis** |
| --- | --- | --- | --- | --- |
|  |  |  |  |  |
|  |  |  |  |  |

L. Occupational disease Yes No (If the answer is “Yes”, please fill the attached questionnaire 6.)

M. Pneumocomosis Yes No (If the answer is “Yes”, please fill the attached questionnaire 7.)

**Questionnaire 6 of the occupational disease history**

| **L1**  **Name of the occupational disease** | **L2**  **Time of Diagnosis** | **L3**  **Work unit** | **L4**  **Reason** | | | **L5**  **Unit of diagnosis** | **L6**  **State of illness** | **L7**  **Notes** |
| --- | --- | --- | --- | --- | --- | --- | --- | --- |
|  |  |  |  |  |  |  |  |  |
|  |  |  |  |  |  |  |  |  |

(Remark: No occupational radioactive disease and pneumoconiosis included.)

**Questionnaire 7 of workers' pneumoconiosis**

| **M1**  **Time of Diagnosis** | **M2**  **Stage of Pneumoconiosis** | **The complication** | | | | | **M7**  **Unit of diagnosis** |
| --- | --- | --- | --- | --- | --- | --- | --- |
|  |  | **M3**  **Tuberculosis** | **M4**  **Pulmonary and bronchial infections** | **M5**  **Spontaneity pneumothorax** | | **M6**  **Pulmonary heart disease** |  |
|  |  |  |  | |  |  |  |
|  |  |  |  | |  |  |  |
|  |  |  |  | |  |  |  |
